# Clinical research perspective on moxibustion treatment for urinary incontinence: A perspective review

**DOI:** 10.1097/MD.0000000000040127

**Published:** 2024-10-11

**Authors:** Xi-Wen Yu, Cheng-Si Wang, Jia-Mei Wu

**Affiliations:** a Department of Acupuncture and Moxibustion, Baicheng Medical College, Baicheng, China; b College of Mathematical Sciences, Shanghai Jiaotong University, Shanghai, China; c School of Basic Medicine, Baicheng Medical College, Baicheng, China.

**Keywords:** efficacy, mechanism, moxibustion, perspective, safety, urinary incontinence

## Abstract

This study provides an in-depth perspective of moxibustion as a treatment option for urinary incontinence (UI), focusing on its clinical efficacy, underlying mechanisms, and potential integration into standard care practices. Moxibustion, rooted in traditional Chinese medicine, involves the targeted application of heat from burning moxa at specific acupoints. Analyzing data from randomized controlled trials and retrospective studies, the study suggests that moxibustion effectively reduces UI symptoms and improves quality of life with minimal adverse effects. The therapeutic benefits are attributed to enhanced blood circulation, improved neurological functions, and hormonal balance, facilitating tissue repair, and urinary system functionality. Despite encouraging outcomes, existing research exhibits limitations, including small sample sizes, and inconsistent methodologies. Future research should aim to address these gaps by conducting larger, standardized multicenter trials to provide more definitive evidence of moxibustion’s effectiveness. Additionally, integrating moxibustion into comprehensive treatment strategies for UI and promoting its inclusion in clinical guidelines could enhance its acceptance and application in modern medical practice. This study underscores the potential of moxibustion as a non-alternative in the management of UI, warranting further exploration and validation in clinical settings.

## 1. Introduction

Urinary incontinence (UI), defined as involuntary urine leakage due to impaired bladder control, poses significant challenges to affected individuals.^[1]^ It disrupts physical comfort and significantly undermines psychological well-being and social engagement.^[1]^ The World Health Organization estimates that around 200 million individuals worldwide experience UI, with a notable prevalence among postpartum and postmenopausal women, highlighting a major public health concern.^[2,3]^

Moxibustion, a traditional therapeutic practice from Chinese medicine, involves the targeted application of heat from burning moxa at specific acupoints.^[4]^ This technique aims to restore balance within the body’s *Yin* and *Yang* and regulate *Qi* (vital energy) and *Blood* flow, thus promoting health and disease prevention.^[4]^ Recent clinical studies suggest that moxibustion may improve UI by enhancing local blood circulation, optimizing neurological function, and modulating hormonal activity.^[5,6]^ These mechanisms are proposed to support tissue repair and functional improvement in the urinary system.^[6]^

Despite its historical use, moxibustion’s role in modern healthcare, particularly for treating UI, necessitates a critical examination through a systematic review.^[6]^ The existing literature is fragmented and varies in methodological quality, with inconsistent findings that complicate the clinical applicability of moxibustion.^[6,7]^ Furthermore, the complex pathophysiology of UI and the multifaceted mechanisms through which moxibustion might exert its effects call for a more detailed scientific exploration.^[5,6]^

This study aims to rigorously analyze existing clinical research and theoretical frameworks regarding moxibustion’s use in UI treatment. It seeks to clarify the therapy’s efficacy, safety, and potential mechanisms of action, offering evidence-based insights for future research and clinical practice. This synthesis will contribute to a better understanding of moxibustion’s potential benefits and limitations, supporting its integration into contemporary treatment paradigms for UI.

## 2. Overview of treatments for UI

**Figure 1.**
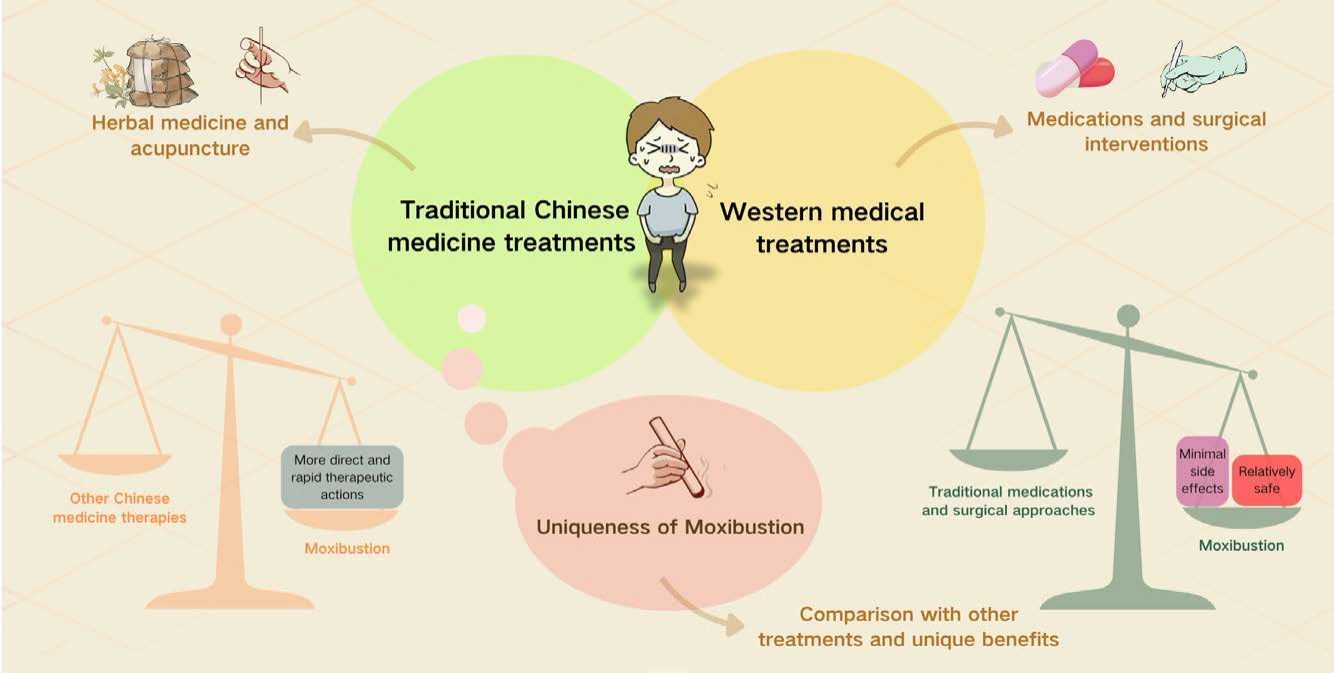
Treatments for urinary incontinence.

The treatment landscape for UI is diverse, encompassing both Western and traditional Chinese medical approaches (Fig. 1). Effective management not only improves physiological conditions but significantly enhances the quality of life for patients.

### 2.1. Western medical treatments: medications and surgery

In Western medicine, UI is primarily treated through pharmacological and surgical interventions (Fig. 1). Pharmacological treatments typically involve anticholinergic drugs to reduce bladder overactivity and hormone replacement therapy, particularly for women, to address incontinence stemming from hormonal imbalances.^[8,9]^ Surgical options include sling procedures and artificial sphincter implants, which are generally considered for patients who do not respond adequately to medications.^[10,11]^ These treatments are widely utilized in clinical settings; however, they can carry various side effects such as dry mouth, constipation, and potential surgical risks.

### 2.2. Traditional Chinese medical treatments: herbal medicine and acupuncture

Traditional Chinese medicine (TCM) addresses UI by focusing on the balance of *Qi* (energy) and *Blood* and strengthening kidney function, which are believed to be closely related to the etiology of UI according to TCM principles.^[12]^ Herbal treatments usually involve a combination of multiple herbs aimed at improving organ function and reducing episodes of incontinence^[13]^ (Fig. 1). Acupuncture involves stimulating specific acupoints to regulate organ function and local blood circulation^[7,14,15]^ (Fig. 1). These methods are considered noninvasive with fewer side effects, though their effectiveness can vary significantly among individuals and generally require stronger scientific validation.

### 2.3. The uniqueness of moxibustion and its comparison with other treatments

Moxibustion, a special form of acupuncture, involves the burning of moxa (dried mugwort) on specific acupoints to deliver a warming stimulus^[4]^ (Fig. 1). This thermal stimulation is thought to penetrate deep into the skin, reaching muscle layers to enhance blood circulation, increase metabolic activity in tissues, and potentially strengthen muscle and nerve functions, which are crucial for treating UI.^[5,6,16]^ Compared to traditional medications and surgical approaches, moxibustion offers a relatively safe alternative with minimal side effects, making it particularly suitable for patients seeking non-pharmacological treatments.^[16]^ Moreover, compared to other TCM therapies, moxibustion provides more direct and rapid therapeutic actions, particularly in improving symptoms of UI.

Moxibustion offers distinct advantages over traditional acupuncture due to its additional thermal effects, which enhance therapeutic outcomes beyond the acupoint stimulation achieved with needles.^[5,17]^ The application of heat from burning moxa not only stimulates the acupoints but also promotes vasodilation, thereby increasing local blood flow.^[5,6]^ This improved circulation delivers more oxygen and nutrients to the affected tissues, which is particularly beneficial in treating UI, as enhanced blood flow can support muscle function and nerve repair in the pelvic region.^[6]^ Furthermore, moxibustion has been shown to activate heat shock proteins (HSPs), which are critical for cellular protection and repair mechanisms.^[4]^ HSPs assist in stabilizing proteins, preventing cellular damage, and promoting tissue recovery—effects that are not typically observed with needle-based acupuncture.^[4]^ Moreover, the heat generated by moxibustion may also modulate the local immune response by reducing levels of pro-inflammatory cytokines, such as, tumor necrosis factor-alpha (TNF-α), Interleukin-1 beta (IL-1β), and Interleukin-6 (IL-6).^[18]^ This reduction in inflammation can alleviate chronic inflammatory conditions affecting the bladder and surrounding tissues. Thus, by combining the mechanical stimulation of acupuncture with the added benefits of heat, moxibustion provides a more comprehensive treatment approach for UI, addressing both symptomatic relief and underlying pathophysiological mechanisms more effectively than acupuncture alone.

In summary, the treatment of UI should be tailored to the specific circumstances and preferences of the patient, as each method presents unique advantages and limitations. As a complementary and alternative therapy, moxibustion offers a valuable option that can be integrated with other modalities to achieve optimal therapeutic outcomes.

## 3. Theoretical foundations and practical applications of moxibustion for UI

Moxibustion, a TCM modality, utilizes the heat from burning dried mugwort to treat various ailments, including UI.^[4]^ This treatment combines TCM principles with insights from modern biomedical research to address both the symptoms and underlying causes of UI.

### 3.1. TCM perspective on UI and moxibustion’s role

In TCM, UI is often attributed to weaknesses in the kidney and bladder’s *Qi* (vital energy), which leads to compromised urinary retention.^[4]^ Moxibustion is believed to reinforce the *Qi* of the kidney and bladder, enhancing their functionality and energy flow, critical for controlling urination.^[4–6]^ The treatment targets specific acupoints linked energetically to the urinary system, aiming to correct the *Qi* deficiencies that contribute to UI.

### 3.2. Specific moxibustion techniques: acupoint selection, duration, and frequency

The selection of acupoints is crucial for effective moxibustion treatment. Key points for UI include Qihai (CV 6), Zhongji (CV 3), and Guanyuan (CV 4), all located on the lower abdomen and associated with urinary health.^[6,19]^ Treatment involves placing a moxa stick near the skin, allowing deep heat penetration.^[4]^ Typical sessions may last between 5 and 15 minutes per acupoint and occur once or twice weekly, depending on symptom severity and patient response.^[6,19]^

Heat-sensitive moxibustion is a specialized form of moxibustion that involves detecting specific acupoints or areas on the body that show a heightened thermal response during treatment.^[20]^ These areas, identified as heat-sensitive points, are thought to reflect underlying energetic imbalances, such as *Qi stagnation* or *deficiencies*, which contribute to the pathophysiology of UI.^[20]^ The application of moxa near these points is believed to enhance the therapeutic effects by precisely targeting these imbalances, leading to more effective regulation of bladder function.^[20]^ This technique is distinguished by its ability to fine-tune the stimulation of acupoints, thereby optimizing the activation of physiological pathways involved in autonomic regulation and bladder control. Research suggests that heat-sensitive moxibustion may improve clinical outcomes in UI management compared to standard moxibustion, as it customizes the treatment to the patient’s specific energetic needs, potentially enhancing the modulation of neural pathways and reducing involuntary bladder contractions. The precise focus on heat-responsive areas allows for a more personalized therapeutic approach, potentially leading to greater improvements in symptom management and overall efficacy.

### 3.3. Mechanisms of moxibustion: integrating biomedical and TCM theories

Biomedically, moxibustion is thought to stimulate blood circulation and enhance local tissue metabolism, aiding in strengthening the muscles and nerves crucial for urinary retention.^[4]^ The heat may also trigger endorphin release, providing natural pain relief, and promote an anti-inflammatory response, potentially alleviating underlying inflammation associated with UI.^[4]^ Moxibustion’s therapeutic effects on UI may be attributed to several underlying biological and physiological mechanisms.^[4]^ Firstly, the thermal stimulation from moxibustion is thought to activate HSPs, which play a crucial role in cellular protection and repair processes.^[4]^ HSPs help stabilize cellular proteins, prevent apoptosis, and facilitate recovery from thermal stress, thereby contributing to the maintenance of bladder tissue integrity.^[4]^ Additionally, moxibustion may influence neurotrophic factors, such as nerve growth factor (NGF), which supports the growth and repair of nerve tissues.^[21]^ Enhanced NGF levels could improve neuromuscular function, thus strengthening pelvic floor muscles and bladder control, which are essential in reducing UI symptoms. Furthermore, moxibustion is believed to modulate the autonomic nervous system by enhancing parasympathetic activity, which promotes bladder relaxation and mitigates involuntary contractions that often contribute to UI.^[20]^ This autonomic regulation helps stabilize bladder function, providing better control over urinary retention. Lastly, moxibustion’s anti-inflammatory effects are linked to its ability to modulate cytokine levels, specifically by downregulating pro-inflammatory cytokines such as TNF-α, IL-1β, and IL-6.^[18]^ These cytokines are commonly elevated in inflammatory conditions affecting the pelvic region, and their reduction can alleviate chronic inflammation, thereby improving the functional capacity of the bladder and associated musculature.

Moxibustion’s effects on UI can be further understood through the exploration of specific molecular pathways and genetic markers that may predict treatment response. Thermal stimulation from moxibustion is thought to activate HSPs, such as HSP70, which protect cells from damage by preventing protein aggregation, stabilizing cellular structures, and enhancing cellular recovery processes.^[4]^ HSPs also play a role in modulating immune responses, including the downregulation of pro-inflammatory cytokines like TNF-α, IL-1β, and IL-6, which are implicated in chronic inflammation of the pelvic region affecting bladder function.^[18]^ Additionally, moxibustion may upregulate neurotrophic factors, such as NGF, which are essential for nerve growth, repair, and the regeneration of pelvic floor muscles, thus improving neuromuscular control of the bladder.^[21]^ Moreover, emerging evidence suggests that moxibustion could influence the expression of genes associated with oxidative stress and apoptosis, promoting tissue repair and enhancing overall bladder health. Identifying genetic or molecular biomarkers linked to these pathways could offer a personalized approach to moxibustion, allowing clinicians to predict which patients are most likely to benefit from the treatment. This detailed exploration not only enriches the scientific basis of moxibustion but also supports its integration into evidence-based clinical practice by providing insights into the mechanisms underlying its therapeutic effects. By integrating these pathways, moxibustion offers a multifaceted approach that addresses both the symptoms and underlying causes of UI, providing a robust scientific basis for its clinical application.

From a TCM standpoint, moxibustion is considered to warm and invigorate *Qi* and *Blood* flow, thereby strengthening kidney and bladder functions.^[4]^ Enhancing *Qi* circulation through these organs helps restore the body’s natural urine control and maintain bladder integrity.

## 4. Clinical research overview on moxibustion for UI

Clinical investigations into moxibustion for UI utilize a range of methodological approaches to assess the efficacy and safety of this traditional Chinese medical intervention. This overview synthesizes study designs, results, and evidence reliability, which are critical for evaluating the clinical applicability of moxibustion.

### 4.1. Assessment of research design

The majority of research on moxibustion for UI comprises randomized controlled trials (RCTs) and retrospective studies^[19,20,22–31]^ (Table 1). RCTs are particularly valuable for their ability to reduce bias through randomization and the use of control groups. These trials frequently juxtapose moxibustion against no intervention, placebo treatments, or conventional medical therapies, thus clarifying its specific therapeutic contributions. For example, recent trials have explored the efficacy of moxibustion alongside Kegel exercises, electroacupuncture, and other modalities across varied clinical environments^[20,22–24,27,28]^ (Table 1). Retrospective study complements these findings by offering insights into the practical implementation and sustained impacts of moxibustion, thereby enriching our understanding of its utility in real-world settings.

**Table 1:** Clinical trial summary of moxibustion for urinary incontinence.

Study Ref.	Disease	Treatment	Publication type	Sample size	Main findings
Jiang 2020^[19]^	UI	Moxibustion and EA	RCT	60	EA, moxibustion, and traditional Chinese medicine effectively treat poststroke UI caused by kidney-*Yang* deficiency
Hu 2017^[20]^	UI	Moxibustion and KET	RCT	45	Heat-sensitive moxibustion plus KET are more effective than KET alone for female stress UI
Tang 2009^[22]^	UI	Moxibustion, Acupuncture and PFME	RCT	71	Acupuncture, tortoise-shell moxibustion, and PFME improve symptoms and quality of life in women with stress UI
Qiao 2022^[23]^	UI	Moxibustion and PFME	RCT	60	Bai Xiao moxibustion combined with PFME is more effective than PFME alone in treating mild to moderate female stress UI
Sun 2022^[24]^	UI	Moxibustion	RCT	60	Herbal moxibustion significantly improves leakage volume, frequency, and life quality in female stress UI patients, outperforming starch-separated moxibustion
Liu 2021^[25]^	UI	Moxibustion	RCT	60	Moxibustion at Baliao acupoints significantly enhances urinary control and reduces infections in poststroke patients, outperforming traditional catheterization
Li 2019^[26]^	UI	Moxibustion and PFME	RCT	60	Moxibustion combined with PFME is effective in treating stress UI in elderly women
Jiang 2019^[27]^	UI	Moxibustion and PFME	RCT	60	PFME combined with moxibustion at the Guanyuan acupoint is an effective method for treating female stress UI
Wang 2018^[28]^	UI	Moxibustion, exercise, and ES	RCT	60	Moxibustion with exercise and biofeedback ES significantly improves and sustains relief of mild to moderate stress UI in women
Chen 2017^[29]^	UI	Moxibustion, biofeedback, and ES	RCT	120	Combining moxibustion with biofeedback and ES is more effective for treating stress UI than PFME alone
Bao 2016^[30]^	UI	Moxibustion	RCT	90	Heat-sensitive moxibustion can reduce the severity of UI in stroke patients and improve their daily living activities
Yan 2015^[31]^	UI	Moxibustion	Retrospective study	43	Combining herbal moxibustion with targeted nursing interventions can significantly improve recovery and psychological well-being in poststroke UI patients

EA = electroacupuncture, ES = electrical stimulation, KET = Kegel exercise therapy, PFME = pelvic floor muscle exercise, RCT = randomized controlled trial, UI = urinary incontinence.

### 4.2. Key findings and data analysis: efficacy and safety

Empirical evidence from RCTs generally supports the effectiveness of moxibustion in ameliorating symptoms of UI, including a decrease in the frequency of incontinence episodes and an improvement in quality of life^[19,25,26,29,30]^ (Table 1). Reports indicate that moxibustion is well-tolerated by patients, with the most common side effect being minor and temporary skin irritation. Observational studies corroborate these findings and suggest that moxibustion may offer enduring benefits, making it a potentially vital option for long-term management of UI^[20,22–24,28,29,31]^ (Table 1).

### 4.3. Evaluation of research quality and result reliability

Variability in the methodological quality of studies poses a notable challenge, as some exhibit robust experimental designs and transparent reporting, while others fall short in critical areas such as allocation concealment and blinding, which are essential for minimizing bias^[19,25,26,29,30]^ (Table 1). Systematic reviews and meta-analyses in this domain suggest that studies with higher methodological quality tend to report more modest effects, pointing to potential overestimations of treatment efficacy in less rigorously conducted studies. The credibility of findings is further influenced by sample size and participant diversity, with larger and more diverse study populations tending to yield more robust and generalizable results^[20,22–24,27–29,31]^ (Table 1).

This analysis underscores the complex yet promising potential of moxibustion as an integrative therapy for UI, advocating for its consideration in holistic urinary health strategies.

## 5. Systematic evaluation of moxibustion’s effectiveness for treating UI

To systematically evaluate moxibustion for UI, a comprehensive analysis of clinical data is essential. This analysis should encompass the efficacy, sustainability of treatment effects, and impact on patient well-being. Key aspects include examining statistical improvements, recurrence rates, comparison with other treatments, and evaluation of patient satisfaction and quality of life.

### 5.1. Treatment outcomes

Evaluating the effectiveness of moxibustion involves measuring improvement rates, which reflect reductions in the frequency and severity of UI episodes following treatment, and recurrence rates, which assess how long these improvements persist. Research indicates that improvement rates for moxibustion range from 60% to 80%, highlighting its potential efficacy.^[19,25,26]^ Furthermore, studies suggest that moxibustion may provide better long-term outcomes compared to conventional treatments, emphasizing the importance of including long-term follow-up data in these assessments.^[27,29,30]^

### 5.2. Comparative effectiveness with other treatments

Comparing moxibustion to alternative UI treatments like pharmacotherapy, physical therapy, and electrical stimulation requires a critical assessment of efficacy and patient preference. Studies have demonstrated that moxibustion is either comparable or superior to these treatments, particularly among patients who prefer non-pharmacological options or are ineligible for surgical interventions.^[20,22,23,28]^ It is vital to incorporate detailed analysis of studies comparing these treatments, focusing on methodologies, patient demographics, and specific outcome measures to establish a robust benchmark for moxibustion’s performance.

### 5.3. Patient satisfaction and quality of life

The impact of moxibustion on patient satisfaction and quality of life is typically assessed through patient-reported outcomes and qualitative data. These evaluations consistently show high levels of patient satisfaction, attributed to the noninvasive nature of moxibustion and minimal adverse effects.^[24,27,31]^ The use of standardized instruments like the Incontinence Quality of Life Questionnaire helps quantify these benefits, providing a solid foundation to assess how moxibustion affects aspects of daily living and psychological well-being before and after treatment.^[23,24,31]^

This study offers a more nuanced understanding of moxibustion’s role in treating UI, supporting its inclusion as an effective treatment modality within a comprehensive care framework. This approach ensures a more rigorous and academically sound evaluation, crucial for healthcare practitioners and policy makers considering moxibustion as a viable treatment option.

## 6. Discussion

This section delves into the mechanisms underlying moxibustion for treating UI, evaluates current research limitations, and explores its implications for clinical practice and future investigations.

### 6.1. Potential mechanisms and clinical significance

Moxibustion is posited to exert its therapeutic effects on UI through several mechanisms.^[4]^ Biomedically, it is thought to enhance local circulation and stimulate peripheral nerve endings, potentially strengthening the pelvic floor muscles and stabilizing bladder control.^[32]^ In the realm of TCM, moxibustion is believed to correct *Qi* imbalances, specifically fortifying the *Qi* of the kidney and bladder, which are crucial for urinary function.^[4]^ This dual approach not only targets the symptoms of UI but also addresses its root causes, potentially reducing the need for pharmacological interventions and improving overall patient outcomes.^[4]^

While the study effectively outlines the general mechanisms by which moxibustion may alleviate UI, it is important to consider the role of tumor heterogeneity in influencing treatment outcomes.^[33]^ UI often occurs in patients with diverse underlying conditions, including various tumor types, each with distinct characteristics that can impact therapeutic efficacy. Tumors differ in location, size, histology, and molecular profiles, all of which may affect the bladder and its control mechanisms differently. For example, tumors located in proximity to the bladder or pelvic nerves might disrupt local blood flow or nerve signaling, potentially altering the response to moxibustion.^[33]^ Additionally, variations in tumor biology, such as distinct inflammatory environments or specific molecular markers, could influence how effectively moxibustion modulates local immune responses and other therapeutic pathways. A thorough exploration of these factors would enable a more personalized approach to moxibustion, tailoring treatment to individual patient profiles based on their specific tumor characteristics. Such an approach would not only optimize the clinical outcomes of moxibustion but also expand its applicability across a broader range of patient subgroups, ultimately supporting its integration into comprehensive and individualized treatment regimens for UI.

### 6.2. Research limitations and future directions

Current research into moxibustion for UI, while insightful, is hampered by several limitations. Many studies feature small sample sizes and short follow-up durations, which can compromise the reliability and applicability of the results.^[34]^ Additionally, the lack of standardized treatment protocols and the subjective nature of some outcome measures hinder the objective evaluation of efficacy. To overcome these challenges, future research should aim for larger-scale, multicenter trials with standardized protocols and extended follow-up periods.^[35]^ Comparative studies with other noninvasive treatments are also essential to position moxibustion within the broader therapeutic landscape, helping to determine the most effective treatment modalities for various UI cases.

### 6.3. Practical application and wider adoption

Moxibustion offers a viable noninvasive treatment option for UI, particularly appealing to patients who are either unresponsive to conventional therapies or prefer non-pharmacological approaches.^[32,34]^ To facilitate its broader adoption, educational initiatives targeted at both clinicians and patients are crucial. These programs should detail the benefits and application techniques of moxibustion, ensuring that healthcare providers are proficient in delivering this therapy. Furthermore, integrating moxibustion into clinical guidelines for UI could promote its legitimacy and encourage wider clinical use. Policy support, such as insurance coverage for moxibustion, would also significantly enhance its accessibility and adoption.

## 7. Summary

Moxibustion has shown considerable promise as a treatment for UI, effectively bridging TCM and contemporary therapeutic methodologies. The therapy is generally well-received by patients, with clinical studies supporting its ability to decrease the frequency of incontinence episodes and enhance overall quality of life. Nonetheless, the robustness of these findings could be improved with further research involving larger, more rigorous randomized controlled trials that utilize standardized treatment protocols and extended monitoring periods.

For integration into clinical practice, moxibustion should be considered as part of a comprehensive treatment strategy for UI that is tailored to individual patient needs and circumstances. Ensuring that clinicians are properly trained in moxibustion techniques will be essential for its successful application.

Future research should aim to elucidate the specific mechanisms through which moxibustion exerts its effects on UI, investigate its long-term efficacy and safety, and conduct comparative analyses with other treatment modalities. This expanded evidence base will enhance the understanding of moxibustion’s therapeutic potential and could facilitate its broader adoption in clinical settings, offering an additional, valuable tool for managing UI.

## Author contributions

**Conceptualization:** Xi-Wen Yu, Cheng-Si Wang, Jia-Mei Wu.

**Data curation:** Xi-Wen Yu, Cheng-Si Wang, Jia-Mei Wu.

**Investigation:** Jia-Mei Wu.

**Methodology:** Xi-Wen Yu, Cheng-Si Wang.

**Project administration:** Jia-Mei Wu.

**Resources:** Xi-Wen Yu, Cheng-Si Wang.

**Supervision:** Jia-Mei Wu.

**Validation:** Xi-Wen Yu, Cheng-Si Wang, Jia-Mei Wu.

**Visualization:** Xi-Wen Yu, Cheng-Si Wang, Jia-Mei Wu.

**Writing – original draft:** Xi-Wen Yu, Cheng-Si Wang, Jia-Mei Wu.

**Writing – review & editing:** Xi-Wen Yu, Cheng-Si Wang, Jia-Mei Wu.
